# Effects of Ethanol Extracts of *Origanum vulgare* and *Thymus vulgaris* on the Mycotoxin Concentrations and the Hygienic Quality of Maize (*Zea mays* L.) Silage

**DOI:** 10.3390/toxins14050298

**Published:** 2022-04-22

**Authors:** Gintarė Vaičiulienė, Bronius Bakutis, Jurgita Jovaišienė, Rimvydas Falkauskas, Gediminas Gerulis, Elena Bartkienė, Dovilė Klupšaitė, Jolita Klementavičiūtė, Violeta Baliukonienė

**Affiliations:** 1 Department of Food Safety and Quality, Faculty of Veterinary Medicine, Lithuanian University of Health Sciences, LT-44307 Kaunas, Lithuania; bronius.bakutis@lsmuni.lt (B.B.); jurgita.jovaisiene@lsmuni.lt (J.J.); rimvydas.falkauskas@lsmuni.lt (R.F.); gediminas.gerulis@lsmuni.lt (G.G.); elena.bartkiene@lsmuni.lt (E.B.); violeta.baliukoniene@lsmuni.lt (V.B.); 2 Institute of Animal Rearing Technologies, Faculty of Animal Science, Lithuanian University of Health Sciences, LT-44307 Kaunas, Lithuania; dovile.klupsaite@lsmuni.lt (D.K.); jolita.klementaviciute@lsmuni.lt (J.K.)

**Keywords:** biogenic amines, herbal plant extracts, hygienic quality, maize silage, mycotoxins, *Origanum vulgare*, *Thymus vulgaris*

## Abstract

The aim of this study was to assess the usefulness of oregano (*Origanum vulgare* L.) and thyme (*Thymus vulgaris* L.) extracts to decrease mycotoxin contents and improve the hygienic quality of maize (*Zea mays* L.) silage. Under laboratory conditions, maize silage samples were fermented with oregano (OE), thyme (TE), oregano and thyme mixture (MIX), and two commercial inoculants. After 90 days of fermentation, silos were opened and silage samples were taken for evaluation of mycotoxin concentrations and for hygienic quality analysis: assessment of fermentation parameters, the content of biogenic amines, and microbiological status. It was determined that the mycotoxin concentrations decreased differentially: ochratoxin A concentration was below the detection limit after treatment with the TE and MIX extracts, the lowest zearalenone and deoxynivalenol concentrations were achieved with the OE extract treatment, T-2 toxin concentration was significantly lower after treatment with the TE extract, and HT-2 toxin concentration was lower after treatment with the MIX extract. The lowest content of biogenic amines in maize silage was established with the MIX extract. Concerning the silage hygienic quality, the best results of fermentation parameters and microbiological status were also achieved with the MIX extract. The present study indicated that oregano and thyme herbal plant extracts can be used to decrease mycotoxin concentrations and improve the hygienic quality of maize silage.

## 1. Introduction

Maize silage is one of the most common feedstuffs for dairy cows in Europe [1]. Maize is a crop with high fermentation capacity, allowing its use to obtain a high nutritional value silage, readily consumed by animals [2]. Maize silage is usually used as a source of energy and fiber for dairy cows. Therefore, silage must be produced in a way that improves its nutritional value without compromising the physiology and productivity of the animals. The quality of silage can depend on many factors that can change its expansion and fermentation patterns [3].

Mycotoxins are low molecular weight secondary metabolites, produced as a toxic secondary metabolite by several fungal species belonging to *Aspergillus*, *Penicillium*, *Fusarium*, *Alternaria,* and *Claviceps* genera. Maize and maize products are among the most vulnerable grains, which may be contaminated with toxigenic fungi and one or more mycotoxins [4]. Under favorable environmental conditions, these fungi can invade crops and produce mycotoxins. These toxic compounds can occur before harvest, and their prevalence can be influenced by environmental factors, during or after harvest, which might be related to improper storage of the silage. Aflatoxin B1 (AFB1), zearalenone (ZEA), deoxynivalenol (DON), ochratoxin A (OTA), fumonisin B1 (FB1), and T-2 and HT-2 toxins, due to their prevalence and adverse effects on human and animal health, are considered the most economically significant mycotoxins [5]. In cattle, mycotoxins can affect feed intake, growth rate, inhibit protein synthesis, milk yield, gut barrier integrity, mucin production and the immune system, as well as cause reproductive problems. These compounds are highly toxic and can be harmful to animal health in various ways, causing neurotoxic, cytotoxic, carcinogenic, hepatocarcinogenic, mutagenic, or teratogenic effects [6,7].

Maize has exceptional ensiling properties due to its relatively high dry matter (DM) content, low buffer capacity, and adequate content of water-soluble carbohydrates. During the ensiling process, soluble carbohydrates and proteins are fermented to organic acids, alcohols, and soluble nitrogenous compounds. The formation of acids, mainly lactic acid, leads to a decrease in pH and inhibits the activity of undesirable microorganisms such as *Clostridia* and *Enterobacteria*, thus preserving DM and nutrients. Aerobic deterioration of silage is mainly related to the development of yeasts and molds that remain dormant under anaerobic conditions and multiply rapidly after repeated exposure to air [8]. Whatever the purpose and benefits of ensiling corn grains, the development of spoilage microorganisms such as mold, bacteria, and yeast may lead to undesirable end products (ethanol, ammonia) or even toxic compounds (biogenic amines and mycotoxins). The hygienic quality, fermentation parameters, and aerobic maize silage stability can be improved by inhibiting undesirable microorganisms with appropriate feed additives [9]. 

Biogenic amines (BAs) are low molecular weight organic nitrogen compounds with aliphatic, aromatic, and heterocyclic structures derived from amino acids. Histamine, tyramine, cadaverine, spermine, spermidine, putrescine, and tryptamine are considered to be the most important BAs occurring in feeds. They can be found in all feeds that contain proteins or free amino acids; therefore, they also exist in ensiled feeds. Amines can be formed in both grass (lucerne, clover, and certain grass species) and maize silages [10]. Occurrence of BAs in silage is an indicator of poor silage quality. The concentration of biogenic amines in maize silage depends on crop harvesting and the ensiling processes. BAs can be found in cattle rumen, body tissues, and body fluids in the digestive system of the animals [11]. The formation of BAs is based on proteolysis, a naturally ensiling process consisting of enzymatic decarboxylation of amino acids by the action of plant proteases and peptidases together with enzymes of various lactic acid bacteria such as the *Lactobacillus*, *Enterococcus,* and *Pediococcus*, as well as species of many bacterial genera that may be present in silage such as *Clostridium*, *Escherichia*, *Bacillus*, *Klebsiella*, and *Pseudomonas* [12]. BAs are produced by decarboxylation of tryptophan, arginine, and histidine. During the initial stage of silage fermentation, the formation of BAs can be affected by temperature, decrease in pH, and oxygen availability. Due to bacterial proteolysis, the presence of BAs is associated with a decrease in silage protein content and loss of nutritional value [13]. These compounds can inhibit feed intake, impair gastrointestinal function, cause ketonuria, decrease milk yield, and affect the nervous system and blood pressure of cattle [14].

Various approaches have been developed to control and prevent the content of BAs and mycotoxins as well as improve the hygienic quality of feed. They are categorized as chemical, physical, and microbiological methods. Still, alternative methods are desired to better prevent the formation of biogenic amines, growth of toxigenic fungi, and promote the detoxification or biotransformation of mycotoxin residues to less toxic or non-toxic forms. Botanicals such as essential oils (EOs), spices, herbs, and their extracts are generally considered to be environmentally friendly and safer alternative sources of bio-agents [15]. Herbal plant extracts are products usually generated from different parts of the plants such as roots, leaves, barks, or seeds. The process usually consists of maceration of the dry (dried) plant material with different polarity solvents such as ethanol, methanol, acetone, or water. Increasingly aromatic plants, such as thyme and oregano, have shown many promising results as a natural feed additive in animal feed due to the presence of a biologically active compound in the herb [16,17]. EOs are mainly composed of hydrocarbons, terpenes, or oxygenated compounds such as aldehydes, alcohols, ketones, oxides, and esters. In nearly every EO are monoterpene compounds, and limonene, α-terpinene, β-myrcene, and camphene are some of the most common. Alcohols found in essential oils such as linalool, citronellol, geraniol, and farnesol possess antiseptic and antimicrobial activity. Phenols found in essential oils normally possess strong antibacterial properties, and thymol, carvacrol, and eugenol were found to be dominant components of oregano and thyme herbal plants [18]. These herbs have been assessed for antioxidant and antimicrobial activities since they are generally recognized as safe (GRAS) by the United States Food and Drug Administration (FDA). Additionally, they have been registered by the European Commission for use as flavoring agents in foodstuffs. Both substances appear to affect cell membrane permeability, resulting in inhibition of vegetative bacterial cell growth and death, but also affecting toxin production [19]. The herbs are also known to benefit animal health including appetite, digestion, and immune system stimulation and antimicrobial, anti-inflammatory, and anti-oxidative action when they are used as feed additives in animal nutrition [20].

The aim of this study was to evaluate and relate the usefulness of oregano (*Origanum vulgare* L.) and thyme (*Thymus vulgaris* L.) extracts with decreased mycotoxin concentrations and improved hygienic quality of maize (*Zea mays* L.) silage. The study hypothesized that phenolic compounds present in ethanol extracts of herbal plants can inhibit mold growth and decrease the formation of secondary metabolites, such as mycotoxins, thereby improving the hygienic quality of maize silage (fermentation parameters, BA formation, and microbiological status).

## 2. Results

### 2.1. Effect of Herbal Plant Extracts on Maize Silage Mycotoxin Concentrations after 90 Days of Fermentation

Table 1 presents OTA, ZEA, DON, T-2 toxin, HT-2 toxin, AFB1, AFB2, AFBG1, and AFBG2 concentrations detected in silage samples after 90 days of ensiled fermentation under laboratory conditions.

The maize silage samples were mostly contaminated with ZEA, DON, T-2 toxin, and HT-2 toxin mycotoxins. In ensiled maize samples, OTA concentrations were below detection limits in C samples and samples treated with the TE and MIX extracts while the lowest OTA concentration was detected in silage treated with JC commercial inoculant, which was a significant decrease of 66.7% compared to CE samples. Assessment of ZEA and DON concentrations yielded their lowest values with the OE extract, which was a significant decrease of 51.65% and 12.67%, respectively, compared to C samples. The concentration of T-2 toxin was lower by 80.63% compared to CE samples established with the TE extract. The lowest HT-2 toxin concentrations in maize silage samples were in the MIX extract treatments, which was a significant decrease of 59.35% compared to CE samples. In all estimated maize silage samples, the AFB1, AFB2, AFG1, and AFG2 concentrations were confirmed to be under the detection limit.

### 2.2. Effect of Herbal Plant Extracts on Maize Silage Fermentation Parameters after 90 Days of Fermentation

The fermentation parameters in maize silage samples after 90 days of fermentation are shown in Table 2.

DM losses in maize silage samples varied from 1.67% to 10.67%. There were no significant differences of the DM and pH parameters in maize silage samples treated with either plant extracts.

Considering the evaluation of acetic, propionic, butyric, and lactic acid contents, the highest values of acetic acid was determined in the TE treatments, which was a significant increase of 38.92% compared to CE samples. In silage samples treated with the OE and TE extracts, similar concentrations of propionic acid were determined; the increase was significant at 2.50- and 2.08-fold, respectively, compared to C and CE samples. The highest content of propionic acid was determined in silage treated with JG commercial inoculant and the increase was significant at 2.8-fold, compared to C samples. The highest content of butyric acid was established in samples treated with the TE and MIX extracts. Its increase was significant at 2.0-fold, compared to CE samples. The highest content of lactic acid was determined in the C samples. In silage treated with the MIX extract, the content of lactic acid, which is beneficial for successful fermentation, was significantly higher (by 77.53%), than in the CE samples.

### 2.3. Effect of Herbal Plant Extracts on Maize Silage Biogenic Amines Content after 90 Days of Fermentation

The content of BAs in maize silage after 90 days of fermentation is presented in Table 3. Among all evaluated maize silage samples, tryptamine and spermin were not detected.

The lowest concentration of phenylethylamine, putrescin, cadaverine, histamine, and spermidine in the maize silage samples was determined in the MIX extract treatment, while the lowest concentration of tyramine was established in the OE extract treatment. The amount of phenyltamine, putrescin, cadaverine, and spermidine in samples treated with the MIX extracts was significantly lower, by 39.79%, 18.76%, 8.33%, and 37.67%, respectively, compared to CE samples. Whereas the concentration of tyramine was significantly lower in samples treated with the OE extract, by 15.10%, compared to CE samples. There were no significant differences in the histamine amounts in maize silage samples treated with the herbal plant extracts compared to controls. 

### 2.4. Effect of Herbal Plant Extracts on Maize Silage Microbiological Population after 90 Days of Fermentation

Results from the microbiological population in maize silage samples after 90 days of fermentation, ensiled under laboratory conditions, are shown in Table 4.

Evaluation of the microbiological population in silage samples yielded the lowest total counts of aerobic bacteria and yeast in samples treated with the MIX extract. Total count of aerobic bacteria was significantly lower, 11.04%, compared to CE samples. Meanwhile, the yeast cell count was significantly lower, 47.39%, compared to the C samples which achieved the highest count of these microorganisms. Molds were not detected in the C samples or in the samples treated with the JC and JG commercial inoculants. The lowest mold number was achieved by using the TE extract. The decrease was significant, 20.97%, compared to CE samples. Among all the maize samples, the highest total count of mesophilic lactic acid bacteria was determined in the C and CE samples and samples treated with the JC commercial inoculant. On the other hand, in samples treated with the MIX extract, the mesophilic lactic acid bacteria count decreased significantly, 16.73%, compared to CE samples.

## 3. Discussion

In the current study, we hypothesized that oregano and thyme herbal plants extracts can decrease the mycotoxin concentrations and improve the hygienic quality of maize silage.

High-quality silage should be a stable feed free of undesirable compounds that can adversely affect the health and productivity of dairy cows [21]. Currently, physical methods including chemical and microbial additives are most commonly used to control or eliminate pathogens in maize silages, because they are not harmful to animal health and contribute to environmental care [22]. In our experiment, we analyzed herbal plant extracts because of their antimicrobial and antioxidant properties as potential sources of safer feed additives. The antimicrobial and antioxidant activity of the various plant components (such as phenolic compounds) varies depending on the chemical structures and concentrations of the active substances [23]. Therefore, in the production of herbal plant extracts as extraction solvents, various concentrations of ethanol are commonly used because of their highest extraction yield. In order to better understand and evaluate their activity, we chose ethanol extracts of oregano and thyme with which we treated the raw maize material before ensiling. In the present study, total phenolic content (TPC) in the TE, OE, and MIX extracts was found to be, 35.34 mg GAE/g, 190.34 mg GAE/g, and 138.54 mg GAE/g, respectively. Similar TPCs of *Thymus vulgaris* L. and *Origanum vulgare* L. ethanol extracts—32 mg GAE/g and 180 mg GAE/g, respectively—were reported by Armatu et al. [24]. However, in the study reported by Ulewicz-Magulska and Wesolowsk [25], the TPC of *Origanum vulgare* L. extracts ranged between 104.3–325.1 mg GAE/g and of *Thymus vulgaris* L., between 74.52–163.10 mg GAE/g. Most likely, this difference could be attributed to variation in plant properties depending on location. 

Specific inhibitors naturally present in spices and herbs have also been shown to inhibit the formation of BAs [26]. Components of plants inhibitors such as thymol, which is a phenolic monoterpene found naturally in EO and extracts, has antioxidant and antimicrobial properties. Thymol and other terpenes are also major components of thyme and oregano herbs [27]. From our data, we could infer that the lowest concentrations of BAs in maize silage samples were found in samples treated with the MIX extract. This extract decreased most of the investigated BAs, except tyramine (*p* < 0.05).

Contamination of feed with mycotoxins is difficult to control due to their low molecular weight and the fact that they are non-immunogenic, thermostable, and have a broad spectrum of toxicity [28]. The plant-based active ingredients have antibacterial and antioxidant activity and strong organoleptic properties, and thus can be effective in pre- and post-harvest control [29]. According to the literature, AFB1, AFB2, AFG1, and AFG2 mycotoxins are not considered a major problem in many European countries; however, ZEA and DON mycotoxins are found in maize silage in increasingly higher quantities. In our study, we obtained similar results. In all maize silage samples treated with herbal plant extracts, mycotoxin concentrations for all aflatoxins tested were below the detection limit, while the highest concentrations obtained were for ZEA and DON. Our research results indicate that the lowest OTA concentrations (*p* < 0.05) were determined in TE and MIX extract-treated samples, and ZEA and DON concentrations (*p* < 0.05) in samples with OE extracts. TE extract in maize silage samples significantly decreased T-2 toxin concentration (*p* < 0.05), while HT-2 toxin concentration (*p* < 0.05) was reduced by the MIX extract. Similarly, Safari et al. [30] also found that selected herbal plant extracts could control and decrease mycotoxin production in feedstuffs.

Various studies have focused on the antioxidant and antimicrobial properties of several oregano and thyme species as an alternative to chemical preservatives [31,32]. From our study, we can also report that the best microbiological integrity of silage samples was obtained with the MIX extract. The lowest total count of aerobic bacteria, count of yeast cells (*p* < 0.05), and total count of mesophilic lactic acid bacteria (*p* < 0.05) were established in maize silage samples treated with the MIX extract. In agreement with Kung and co-authors [21], the yeast and mold counts in silages may be useful indicators of ethanol concentration, since a high number of yeast cells is usually associated with a high concentration of ethanol, and their numbers are often inversely related to the aerobic stability of silages. This is especially characteristic in corn-based crops. According to Val de Assis et al. [33], yeast population may decrease during the silage fermentation. This may be due to substrate restriction and/or the formation of metabolites that may inhibit these microorganisms. Yeasts are facultative aerobic microorganisms that are able to survive in environments with low oxygen concentration; they can change their metabolism to prioritize fermentation, and as a result, the number of cells does not increase.

Producing high-quality silage while avoiding DM loss as much as possible is a big challenge. The DM losses and silage quality changes can occur at all stages of the ensiling process, especially at the fermentation stage, thus reducing the quality of feed [34]. In our study, the content of DM in maize samples varied from 30.33% to 39.33% and was considered adequate for the ensiling process. All maize silage samples treated with herbal plant extracts showed a typical fermentation pattern and extremely low DM loss, which is expected when using natural feed additives. In the analyzed samples, pH values ranged from 3.92 to 4.15, which is considered in the literature to be adequate for maize silage. In this study, the best results for fermentation parameters were also achieved with the MIX extract treatment. The ratio of lactic acid to acetic acid is usually used as a qualitative indicator of silage fermentation. In good silage fermentation, the ratio of these acids is typically 2.5–3.0. Silages with extremely low levels of lactic acid and acetic acid ratios may be more aerobically unstable than those with normal ratios because low concentrations of acetic acid may not be sufficient to inhibit lactate-assimilating yeasts. A ratio of lactic to acetic acid of less than one usually indicates abnormal fermentation [21]. In our study, we established similar results; in maize silage samples treated with herbal plant extracts, the lactic acid to acetic acid ratio was between 2.39 to 3.43.

Therefore, our results indicate that ethanol extracts of *Origanum vulgare* L. and *Thymus vulgaris* L. could improve maize (*Zea mays* L.) silage fermentation indicators. In addition, our research results highlight the potential of plant-based bioactive compound use in plant and silage protection.

## 4. Conclusions

According to the results of our study, our findings showed differential effects of the plant extract treatment on different mycotoxin concentrations: OTA concentration was determined to be below the detection limit after treatment with the TE and MIX extracts, the lowest ZEA and DON concentrations were achieved with the OE extract, T-2 toxin concentration was significantly lower after the TE extract treatment, and HT-2 toxin concentration decreased with the MIX extract. Based on the obtained results, it could be concluded that the best results of fermentation parameters and microbiological status were established with the MIX extract. For the content of BAs in maize silage samples ensiled under laboratory conditions, the best results were also achieved with the MIX extract. Additionally, further studies are required using active substances of the herbal plant extracts as well as larger number of samples to investigate their mode of action in maize silage. The results of our study indicate that ethanol extracts of oregano and thyme can be very useful in maize silage production practices to decrease the content of BAs and mycotoxin concentrations and improve the overall hygienic quality of maize silage.

## 5. Materials and Methods

### 5.1. Maize Material

The whole plant maize (*Zea mays* L., cultivar *Auxxel FAO 190*) used in this experiment was harvested at the kernel half-milk line and collected from a dairy farm in Lithuania (55°10′24.96″ N 23°53′41.29″ E). The content of BAs and volatile fatty acids, mycotoxin concentrations, DM, pH, and microbiological population of the raw maize material before fermentation are given in Table 5. All samples were analyzed in triplicate.

### 5.2. Preparation of Herbal Plant Extracts

The extracts were prepared from oregano (*Origanum vulgare* L.) and thyme (*Thymus vulgaris* L.) herbal plant material under laboratory conditions. The air-dried aerial parts were obtained from the “Švenčionių vaistažolės” company (Švenčionys, Lithuania). Herbal plant extracts were produced by maceration, using ethanol as the solvent according to Handa et al. [35] and Tayel et al. [36]. The air-dried herbal plants were chopped, and 25 g of individual plant mass was mixed with 200 mL of 50% ethanol, covered, and stored at room temperature for 24 h, avoiding direct solar radiation. After 24 h, the macerate was filtered using Whatman No 1. filter paper, and the obtained herbal plant extracts were immediately inserted into the ensiled maize samples. The obtained herbal plant extracts were: TE—ethanol extract of thyme (25 g *Thymus vulgaris* L.), OE—ethanol extract of oregano (25 g *Origanum vulgare* L.), and MIX—ethanol extract of oregano and thyme mix (12.5 g. *Origanum vulgare* L. and 12.5 g *Thymus vulgaris* L.).

### 5.3. Determination of Total Phenolic Content (TPC)

The TPC of the ethanol-extracted oregano and thyme was determined by colorimetric spectrophotometry following the Follin-Ciocalteu method [37] with some modifications. For the analysis, 1 mL of each herbal plant extract was mixed separately with 5 mL of the Folin-Ciocalteu reagent (diluted ten-fold). After 5 min, 4 mL of Na_2_CO_3_ solution (75 g/L) was added, mixed thoroughly, and allowed to stand for 60 min at room temperature in the dark. The absorbance of extracts was measured at 760 nm and compared to the prepared blank. The TPC was calculated on the basis of the calibration curve of gallic acid and the results were expressed as gallic acid equivalents (GAE), in milligrams per gram of the sample. All samples were analyzed in triplicate. TPCs of the herbal plant extracts are shown in Table 6.

### 5.4. Preparation of Maize Silage Samples Inoculated with Plant Extracts under Laboratory Conditions

Harvested raw maize material was chopped to 1.5–2 cm diameter particles and ensiled in 3 L mini-silos. Then, 20 ml of each herbal plant extract was well mixed with 1 kg of raw mass, separately. Control (C) silage without any treatment and control silage (CE) with 50% ethanol were produced on the same principle. Moreover, silage with two commercial additives (silage inoculants), JC (6 mg/kg), and JG (3 mg/kg) were also produced following the manufacturer’s instructions. The following bacteria were present in the commercial additives: additive JC—*Lactobacillus plantarum*, *Enterococcus faecium*, and *Pediococcus acidilactici* (1.0 × 10^11^ CFU/g) and additive JG—*L. plantarum*, *P. acidilactici* (1.0 × 10^11^ CFU/g), and xylanase. After introducing the herbal plant extracts into the maize mass, silos were hermetically sealed and stored at 20 °C for 90 days. For each plant extract, controls, and commercial additives, three mini-silos were produced. The ensiling under laboratory conditions was performed based on modified Tayel et al. [36] and Jatkauskas et al. [38] methods. After 90 days of maize fermentation, silos were opened and samples (*n* = 21) were taken in order to evaluate the contents of BAs, mycotoxin concentrations, as well as for hygienic quality analysis: assessment of fermentation parameters and microbiological status.

### 5.5. Determination of Mycotoxin Concentrations

The degree of contamination with AFB1, AFB2, AFG1, AFG2, ZEA, OTA, T-2 toxin, and HT-2 toxin of maize samples was tested by high-performance liquid chromatography (HPLC) with fluorescent detector (FLD) (Model LCMS-8060 Shimadzu Corp., Kyoto, Japan) and contamination with DON by HPLC with ultraviolet detector (UV) (Model Sciex API 5000, McKinley Scientific, Sparta Township, NJ, USA). The air-dried silage samples were ground to pass a 1 mm screen and homogenized. Silage samples were extracted in distilled water for DON, in methanol:water (75:25 *v/v*) for AFB1, AFB2, AFG1, AFG2, and ZEA, and in methanol:water (60:40 *v/v*) for T-2 toxin, HT-2 toxin, and OTA under constant agitation on a mechanical shaker (Phoenix Instrument RS-OS 20, Inc, Garbsen, Germany) for 60 min at 23 °C. After extraction, samples were centrifuged at relative centrifugal force (RCF) 3468× *g* for 10 min (Centrifuge MPW-251, MPW, Warsaw, Poland). Subsequently, the supernatants were filtered with PTFE syringe filters with 0.22 μm diameter pores (Millex-GS, Millipore, Billerica, MA, USA) and diluted with phosphate-buffered saline (PBS). For the sample purification step, the extracts were passed through a multi-mycotoxin immunoaffinity column 11 + Myco MS-PREP^®^ (R-Biopharm AG, Pfungstadt, Germany) according to the manufacturer’s recommendations. For the determination of T-2 toxin and HT-2 toxin, derivatization of maize silage samples was performed according to the method described by Kachuei et al. [39] with some modifications. The prepared samples were subjected to high-performance liquid chromatography analysis, the parameters of which are given in Table 7. Chromatographic separation of mycotoxins was performed using a LiChrospher^®^ 100 RP-18, LiChroCART 250-4 column (250 × 4.0 mm, 5 μm; Supelco Park, Bellefonte, PA, USA). Mycotoxin concentrations were determined by comparing peak retention times with standard solutions. The mycotoxin concentrations were determined by correlation of peak area of the samples with the standard curves, obtained by HPLC analysis of standard solutions.

### 5.6. Determination of DM Content and pH

The DM content and DM losses were determined by weight analysis. For DM determination, silage samples were chopped into particles 3–4 cm in diameter and dried at 55 °C for 18 h. After air equilibration, samples were again weighed and dried at 103 °C for 20 h to constant weight [40]. The pH level was measured by electrometric method with a pH meter (WTW^®^inoLab pH 720, Weilheim in Oberbayern, Germany) in diluted samples (10 g silage sample was homogenized with 90 mL of distilled water for 25–30 min.). The pH was determined from the dial reading with an accuracy of at least +/−0.05 pH units once a constant value was found.

### 5.7. Determination of Volatile Fatty Acids (VFA) Content

Sample preparation for VFAs analysis including lactic, acetic, propionic, and butyric acids in maize silage samples was performed according to the method described by Bureenok et al. [41]. The analyses were carried out using gas chromatograph GC-2 Plus (Shimadzu Corp., Kyoto, Japan) with mass spectrometry detector GCMS-QP2010 (Shimadzu Corp., Kyoto, Japan), as described by Bartkiene et al. [42] with some modifications. The volatile compounds adsorbed by the fiber were desorbed in the injection port of the Shimadzu GC, equipped with a Shimadzu GCMSQP2010 ultra mass-selective detector. For the separation of volatile fatty acids, a Stabilwax^®^-DA column (30 × 0.25 mm, 0.25 μm; Restek Corp., Bellefonte, PA, USA) was used. The oven temperature change was programmable from 50 °C to 260 °C. The injection temperature was 200 °C, carrier was gas helium (>99% purity), and flow rate was − 1 mL/min. The content of volatile fatty acids (percent of total acid content) was calculated using a “GC solution” chromatographic data processing program (Shimadzu Corp., Kyoto, Japan).

### 5.8. Determination of BA Content

Sample preparation and determination of BAs including phenylethylamine, putrescin, cadaverine, histamine, tyramine, spermidine, spermine, and tryptamine in maize silage samples were conducted based on Ben-Gigirey et al. [43] with some modifications, as described by Bartkiene et al. [44]. The extraction of BAs from maize samples (5 g of each sample) was performed by using 0.4 mol/L perchloric acid. The standard BA solutions were prepared by dissolving known amounts of each BA (including internal standard) in 20 mL of deionized water. The derivatization of samples and standards was performed using a dansyl chloride solution (10 mg/mL) as a reagent. The chromatographic analyses were carried out using a Varian ProStar HPLC system (Varian Corp., Palo Alto, CA, USA) with two ProStar 210 pumps, a ProStar 410 autosampler, a ProStar 325 UV/VIS detector, and Galaxy software for data processing (Agilent, Santa Clara, CA, USA). For the separation of BAs, a Discovery^®^ HS C18 column (150 × 4.6 mm, 5 μm; Supelco Park, Bellefonte, PA, USA) was used. The eluents were ammonium acetate (A) and acetonitrile (B), and the elution program consisted of a gradient system with a 0.8 mL/min flow rate. The BA detection wavelength was 254 nm and the oven temperature was 40 °C. The injection volume was 20 μL. The target compounds were identified based on their retention times in comparison to their corresponding standards.

### 5.9. Determination of Microbiological Status

The microbiological population of maize silage was determined according to ISO standards: total count of aerobic bacteria (LST EN ISO 4833-1:2013), total count of mesophilic lactic acid bacteria (LST ISO 15214:2009), and total count of yeast and molds (LST ISO 21527-1:2008).

### 5.10. Statistical Analysis

The data were analyzed using IBM SPSS Statistics v26 for Windows (IBM Corp., Armonk, NY, USA). The differences in the test properties of the compared groups were expressed as mean values and standard deviations (SD). The results were expressed as the mean value of at least three measurements. To evaluate the effect of ethanol extracts of herbal plants on the content of BAs, mycotoxin concentrations, and hygienic quality (fermentation parameters and microbiological status) in maize silage, one-way ANOVA analysis was used. The differences between the compared groups were assessed using Fisher’s LSD test (α = 5%). The obtained results were considered to be statistically significant when *p* < 0.05.

## Figures and Tables

**Table 1:** Mycotoxin concentrations in maize silage after fermentation.

Parameters, µg/kg (ppb)	Silage Samples (Mean ± SD)							*p*-Value
	C	CE	OE	TE	MIX	JC	JG	
Ochratoxin A	BDL	0.12 ± 0.02 ^a^	0.30 ± 0.01 ^b^	BDL	BDL	0.04 ± 0.01 ^c^	0.07 ± 0.01 ^d^	<0.001
Zearalenone	534.75 ± 1.70 ^a^	335.55 ± 2.50 ^b^	258.63 ± 0.61 ^c^	1533.47 ± 2.06 ^d^	1403.75 ± 3.40 ^e^	585.34 ± 0.31 ^f^	495.74 ± 0.36 ^g^	<0.001
Deoxynivalenol	3630.18 ± 2.01 ^a^	3517.28 ± 4.40 ^b^	3170.08 ± 1.96 ^c^	4486.11 ± 1.00 ^d^	4878.82 ± 7.51 ^e^	5064.52 ± 3.26 ^f^	6294.08 ± 0.91 ^g^	<0.001
T-2 toxin	14.18 ± 0.03 ^a^	29.90 ± 1.02 ^b^	16.12 ± 0.13 ^c^	5.79 ± 0.22 ^d^	6.76 ± 0.06 ^e^	10.82 ± 0.23 ^f^	16.92 ± 0.08 ^g^	<0.001
HT-2 toxin	482.65 ± 2.12 ^a^	1017.50 ± 0.70 ^b^	472.28 ± 1.11 ^c^	421.19 ± 1.67 ^d^	413.66 ± 4.36 ^e^	475.68 ± 0.20 ^c^	683.87 ± 2.56 ^f^	<0.001
AFB1, AFB2, AFG1, AFG2	BDL	BDL	BDL	BDL	BDL	BDL	BDL	

^a, b, c, d, e, f, g^—means in row marked with different letters differed significantly (*p* < 0.05). C—control sample (without any treatment); CE—control with ethanol (50%); OE—ethanol extract of oregano; TE—ethanol extract of thyme; MIX—ethanol extract of oregano and thyme mix; JC—control with commercial inoculant (*L. plantarum*, *E. faecium*, and *P. acidilactici*); JG—control with commercial inoculant (*L. plantarum*, *E. faecium*, and *xylanase*); BDL—below detection limit; and SD—standard deviation.

**Table 2:** Fermentation parameters in maize silage after fermentation.

Parameters	Silage Samples (Mean ± SD)							*p*-Value
	C	CE	OE	TE	MIX	JC	JG	
Dry Matter (%)	33.0 ± 3.61	39.3 ± 0.58	31.3 ± 0.58	32.3 ± 1.53	30.3 ± 3.06	38.7 ± 3.79	30.3 ± 3.51	0.072
pH	4.06 ± 0.21	4.05 ± 0.10	4.15 ± 0.02	4.01 ± 0.13	3.92 ± 0.19	4.12 ± 0.16	3.97 ± 0.20	0.100
Acetic acid (g/kg)	2.44 ± 0.12 ^a, c, d, g^	1.85 ± 0.06 ^b^	2.43 ± 0.15 ^c^	2.57 ± 0.03 ^d^	2.05 ± 0.08 ^e^	1.94 ± 0.04 ^b, e^	2.31 ± 0.27 ^c, g^	<0.001
Propionic acid (g/kg)	0.10 ± 0.02 ^a^	0.12 ± 0.01 ^a^	0.25 ± 0.01 ^b^	0.25 ± 0.01 ^b^	0.17 ± 0.00 ^c^	0.19 ± 0.01 ^c^	0.28 ± 0.07 ^d^	<0.001
Butyric acid (g/kg)	0.05 ± 0.01 ^a, d, e^	0.08 ± 0.01 ^b^	0.03 ± 0.01 ^c, f^	0.04 ± 0.01 ^c, d, e^	0.04 ± 0.01 ^e^	0.02 ± 0.01 ^f^	0.02 ± 0.02 ^f^	<0.001
Lactic acid (g/kg)	8.11 ± 0.34 ^a^	3.96 ± 0.04 ^b^	6.14 ± 0.19 ^c^	6.15 ± 0.18 ^c^	7.03 ± 0.38 ^d^	6.47 ± 0.54 ^c^	6.21 ± 0.08 ^c^	<0.001

^a, b, c, d, e, f, g^—means in row marked with different letters differed significantly (*p* < 0.05). C—control sample (without any treatment); CE—control with ethanol (50%); OE—ethanol extract of oregano; TE—ethanol extract of thyme; MIX—ethanol extract of oregano and thyme mix; JC—control with commercial inoculant (*L. plantarum*, *E. faecium*, and *P. acidilactici*); JG—control with commercial inoculant (*L. plantarum, E. faecium*, and *xylanase*); and SD—standard deviation.

**Table 3:** Biogenic amines content in maize silage after fermentation.

Parameters (mg/kg DM)	Silage Samples (Mean ± SD)							*p*-Value
	C	CE	OE	TE	MIX	JC	JG	
Phenylethylamine	230.63 ± 16.25 ^a, b, c^	279.08 ± 52.64 ^a^	233.12 ± 8.05 ^a, b, c^	389.03 ± 12.84 ^a^	168.04± 4.54 ^b^	182.19 ± 7.32 ^c, b^	249.06 ± 90.99 ^a, c^	0.010
Putrescin	970.57 ± 163.17 ^a, b^	1003.01 ± 87.77 ^a, b^	1010.00 ± 35.69 ^a, b^	1013.01 ± 87.77 ^a, b^	814.91 ± 127.90 ^a^	1081.74 ± 34.11 ^a, b^	1204.18 ± 338.50 ^b^	0.009
Cadaverine	216.63 ± 62.19 ^a^	292.59 ± 62.50 ^a, b^	278.48 ± 49.94 ^a, b^	257.13 ± 44.18 ^a, b^	268.23 ± 75.31 ^a, b^	311.32 ± 41.74 ^a, b^	467.89 ± 317.24 ^b^	0.035
Histamine	112.44 ± 83.77	133.74 ± 39.30	191.34 ± 62.17	123.74 ± 39.30	112.90 ± 77.35	167.39 ± 17.31	195.76 ± 166.39	0.238
Tyramine	130.52 ± 25.13 ^a^	160.36 ± 8.19 ^b, c^	136.16 ± 7.64 ^c^	180.73 ± 5.16 ^b, c^	154.57 ± 8.42 ^a, b, c^	159.70 ± 1.47 ^b, c^	179.41 ± 27.44 ^b^	0.002
Spermidine	32.38 ± 0.85 ^a, c, d^	45.44 ± 6.10 ^b^	39.51 ± 6.19 ^a, b^	48.66 ± 9.20 ^b^	28.32 ± 0.85 ^c^	28.53 ±1.40 ^c^	40.50 ± 11.49 ^b, d^	0.003

^a, b, c, d^—means in row marked with different letters differed significantly (*p* < 0.05). C—control sample (without any treatment); CE—control with ethanol (50%); OE—ethanol extract of oregano; TE—ethanol extract of thyme; MIX—ethanol extract of oregano and thyme mix; JC—control with commercial inoculant (*L. plantarum*, *E. faecium*, and *P. acidilactici*); JG—control with commercial inoculant (*L. plantarum*, *E. faecium*, and *xylanase*); DM—dry matter; and SD—standard deviation.

**Table 4:** Microbiological populations in maize silage after fermentation.

Parameters (log^10^ CFU/g)	Silage Samples (Mean ± SD)							*p*-Value
	C	CE	OE	TE	MIX	JC	JG	
Total count of aerobic bacteria	4.62 ± 0.17 ^a, b^	4.80 ± 0.27 ^a^	4.58 ± 0.65 ^a, b^	4.92 ± 0.02 ^a, b^	4.27 ± 0.04 ^b^	4.79 ± 0.38 ^a, b^	4.88 ± 0.42 ^a^	0.036
Count of yeast	4.60 ± 0.61 ^a^	4.40 ± 0.36 ^a^	4.38 ±0.35 ^a^	4.33 ± 0.20 ^a^	2.42 ± 1.46 ^b^	3.10 ± 1.93 ^a, b^	4.44 ± 0.74 ^a^	0.019
Count of molds	0.00 ± 0.00 ^a^	2.48 ± 0.79 ^b^	2.15 ± 0.00 ^b^	1.96 ± 1.16 ^b^	2.50 ± 0.47 ^b^	0.00 ± 0.00 ^a^	0.00 ± 0.00 ^a^	<0.001
Total count of mesophilic lactic acid bacteria	5.42 ± 0.03 ^a^	5.32 ± 0.06 ^a, b^	4.55 ± 0.23 ^b, c^	4.97 ± 0.08 ^a, b, c^	4.43 ± 1.16 ^c^	5.35 ± 0.11 ^a^	5.19 ± 0.09 ^a, b, c^	0.018

^a, b, c^—means in row marked with different letters differed significantly (*p* < 0.05). C—control sample (without any treatment); CE—control with ethanol (50%); OE—ethanol extract of oregano; TE—ethanol extract of thyme; MIX—ethanol extract of oregano and thyme mix; JC—control with commercial inoculant (*L. plantarum*, *E. faecium*, and *P. acidilactici*); JG—control with commercial inoculant (*L. plantarum*, *E. faecium*, and *xylanase*); SD—standard deviation.

**Table 5:** Raw maize material before fermentation.

Biogenic Amines (mg/kg DM ± SD)								
Tryptamine	Phenylethylamine	Putrescin		Cadaverine	Histamin	Thyramine	Spermidine	Spermin
0.0 ± 0.0	272.42 ± 27.76	99.54 ± 23.58		63.23 ± 7.95	0.0 ± 0.0	0.0 ± 0.0	63.61 ± 23.90	0.0 ± 0.0
**Mycotoxins (µg/kg ± SD)**								
**OTA**	**ZEA**	**DON**	**T-2 toxin**	**HT-2 toxin**	**AFL B1**	**AFL B2**	**AFLG1**	**AFLG2**
7.09 ± 0.01	556.76 ± 3.27	4608.72 ± 2.09	176.61 ± 0.57	515.13 ± 2.06	BDL	BDL	BDL	BDL
**DM ± SD (%)**	**pH ± SD**	**Volatile Fatty Acids (g/kg DM ± SD)**						
41.0 ± 1.00	5.01 ± 0.05	**Acetic acid**		**Propionic acid**		**Butyric acid**		**Lactic acid**
		0.18 ± 0.01		0.0 ± 0.0		0.01 ± 0.0		0.01 ± 0.01
**Microbiological population (log10 CFU/g ± SD)**								
**Total count of aerobic bacteria**	**Count of yeast**			**Count of molds**		**Total count of mesophilic lactic acid bacteria**		
5.59 ± 0.02	4.92 ± 0.03			3.65 ± 0.60		4.37 ± 0.04		

DM—dry matter; SD—standard deviation; and BDL—below detection limit.

**Table 6:** Total phenolic content of plant extracts.

Extract	Absorbance ± SD	Total Phenolic Content (mg/g GAE)
TE	0.48 ± 0.07	35.34
OE	2.57 ± 0.04	190.34
MIX	0.52 ± 0.06	138.54

TE—ethanol extract of thyme; OE—ethanol extract of oregano; MIX—ethanol extract of oregano and thyme mix; and SD—standard deviation.

**Table 7:** HPLC parameters for the detection of mycotoxins.

Parameters	Mycotoxins				
	AFB1, AFB2, AFG1, AFG2	DON	T-2, HT-2 Toxin	ZEA	OTA
Column temperature	30 °C	30 °C	40 °C	30 °C	30 °C
Mobile phase	H_2_O/ACN/MeOH (60:20:30)	H_2_O/ACN/MeOH (94:3:3)	H_2_O/ACN (40:60)	H_2_O/ACN/MeOH (46:46:8)	H2O/ACN/ACET (46:46:8)
Fluorescent detector, wavelength λ (nm) (excitation and emission)	365 and 435	-	381 and 470	274 and 418	333 and 460
UV detector λ (nm)	-	218	-	-	-
Flow rate (ml/min)	1	1	1	1	1
Injection volume (μL)	100	100	100	100	100
Limit of detection (LOD) (μg/kg)	0.2	20	1.4	3	0.2

## Data Availability

The data presented in this study are available within the article.
